# Construction of machine learning-based models for cancer outcomes in low and lower-middle income countries: A scoping review

**DOI:** 10.3389/fonc.2022.976168

**Published:** 2022-12-01

**Authors:** John Adeoye, Abdulwarith Akinshipo, Mohamad Koohi-Moghadam, Peter Thomson, Yu-Xiong Su

**Affiliations:** ^1^ Division of Oral and Maxillofacial Surgery, Faculty of Dentistry, University of Hong Kong, Hong Kong, Hong Kong, SAR, China; ^2^ Oral Cancer Research Theme, Faculty of Dentistry, University of Hong Kong, Hong Kong, Hong Kong, SAR, China; ^3^ Department of Oral and Maxillofacial Pathology and Biology, Faculty of Dentistry, University of Lagos, Lagos, Nigeria; ^4^ Division of Applied Oral Sciences and Community Dental Care, Faculty of Dentistry, University of Hong Kong, Hong Kong, Hong Kong, SAR, China; ^5^ Clinical Artificial Intelligence Research Theme, Faculty of Dentistry, University of Hong Kong, Hong Kong, Hong Kong, SAR, China; ^6^ College of Medicine and Dentistry, James Cook University, Cairns, Queensland, Australia

**Keywords:** artificial intelligence, cancer, machine learning, low-income countries, lower-middle-income countries

## Abstract

**Background:**

The impact and utility of machine learning (ML)-based prediction tools for cancer outcomes including assistive diagnosis, risk stratification, and adjunctive decision-making have been largely described and realized in the high income and upper-middle-income countries. However, statistical projections have estimated higher cancer incidence and mortality risks in low and lower-middle-income countries (LLMICs). Therefore, this review aimed to evaluate the utilization, model construction methods, and degree of implementation of ML-based models for cancer outcomes in LLMICs.

**Methods:**

PubMed/Medline, Scopus, and Web of Science databases were searched and articles describing the use of ML-based models for cancer among local populations in LLMICs between 2002 and 2022 were included. A total of 140 articles from 22,516 citations that met the eligibility criteria were included in this study.

**Results:**

ML-based models from LLMICs were often based on traditional ML algorithms than deep or deep hybrid learning. We found that the construction of ML-based models was skewed to particular LLMICs such as India, Iran, Pakistan, and Egypt with a paucity of applications in sub-Saharan Africa. Moreover, models for breast, head and neck, and brain cancer outcomes were frequently explored. Many models were deemed suboptimal according to the Prediction model Risk of Bias Assessment tool (PROBAST) due to sample size constraints and technical flaws in ML modeling even though their performance accuracy ranged from 0.65 to 1.00. While the development and internal validation were described for all models included (n=137), only 4.4% (6/137) have been validated in independent cohorts and 0.7% (1/137) have been assessed for clinical impact and efficacy.

**Conclusion:**

Overall, the application of ML for modeling cancer outcomes in LLMICs is increasing. However, model development is largely unsatisfactory. We recommend model retraining using larger sample sizes, intensified external validation practices, and increased impact assessment studies using randomized controlled trial designs

**Systematic review registration:**

https://www.crd.york.ac.uk/prospero/display_record.php?RecordID=308345, identifier CRD42022308345.

## Introduction

An estimated 20 million new cancer cases and 10 million cancer-related deaths occur annually (1). By 2040, the incidence rate is extrapolated to increase by 47% with the highest excess relative risks (64 – 95%) to be observed in low- and middle-income countries. Moreover, mortality estimates are expected to parallel the increase in case occurrence (1). Precision prevention, diagnosis, risk stratification, and treatment are now being advocated as contemporaneous strategies to mitigate the incidence, morbidity, and mortality of patients (2). This involves ultramodern tools and approaches that uncover personalized profiles of patients and tumors, ultimately, assisting in tailoring specific interventions to patients while avoiding the need to administer generic intervention strategies to all individuals (3, 4).

Artificial intelligence (AI) plays a central role in the administration of personalized medicine for cancer patients. Digital systems based on AI can provide objective judgments that simulate human intelligence while considering patient- or tumor-related factors (5, 6). Applications of AI in oncological management include medical/pathological image or video analysis, natural language processing of free-full text electronic health record (EHR) reports, robots, and chatbots as intervention assistants and information resources, affective computing for digital health assistants, automated treatment planning and scheduling, and machine learning (ML) models for the prediction of cancer-related outcomes (7–15). ML-based models have been proposed or evaluated regarding their ability to perform individualized diagnosis, risk stratification, tumor profiling, assisted screening, treatment selection, and disease prognosis prediction (7, 14–18). However, many of the predictive intelligent models have been constructed for and using populations in upper-middle and high-income countries in line with the current distributions of cancer burden (19).

With the expected surge in cancer incidence and mortality among low- and lower-middle-income countries (1), it is imperative to assess the construction and utilization status of ML-based models and platforms in readiness for their implementation in precision cancer interventions in these settings. As external validation of ML-based models available in the Global North may not see fruition in many developing countries owing to the disparities in ethnicity, variability in clinical and molecular cancer subtypes and unavailability of several high-precision advanced cancer predictors, indigenous models developed specifically for these populations may take precedence (19–21). Therefore, in this study, we evaluate the utilization, methodology of model construction, and implementation phases performed for ML-based models for cancer outcomes in low- and lower-middle-income populations.

## Methods

**Figure 1:**
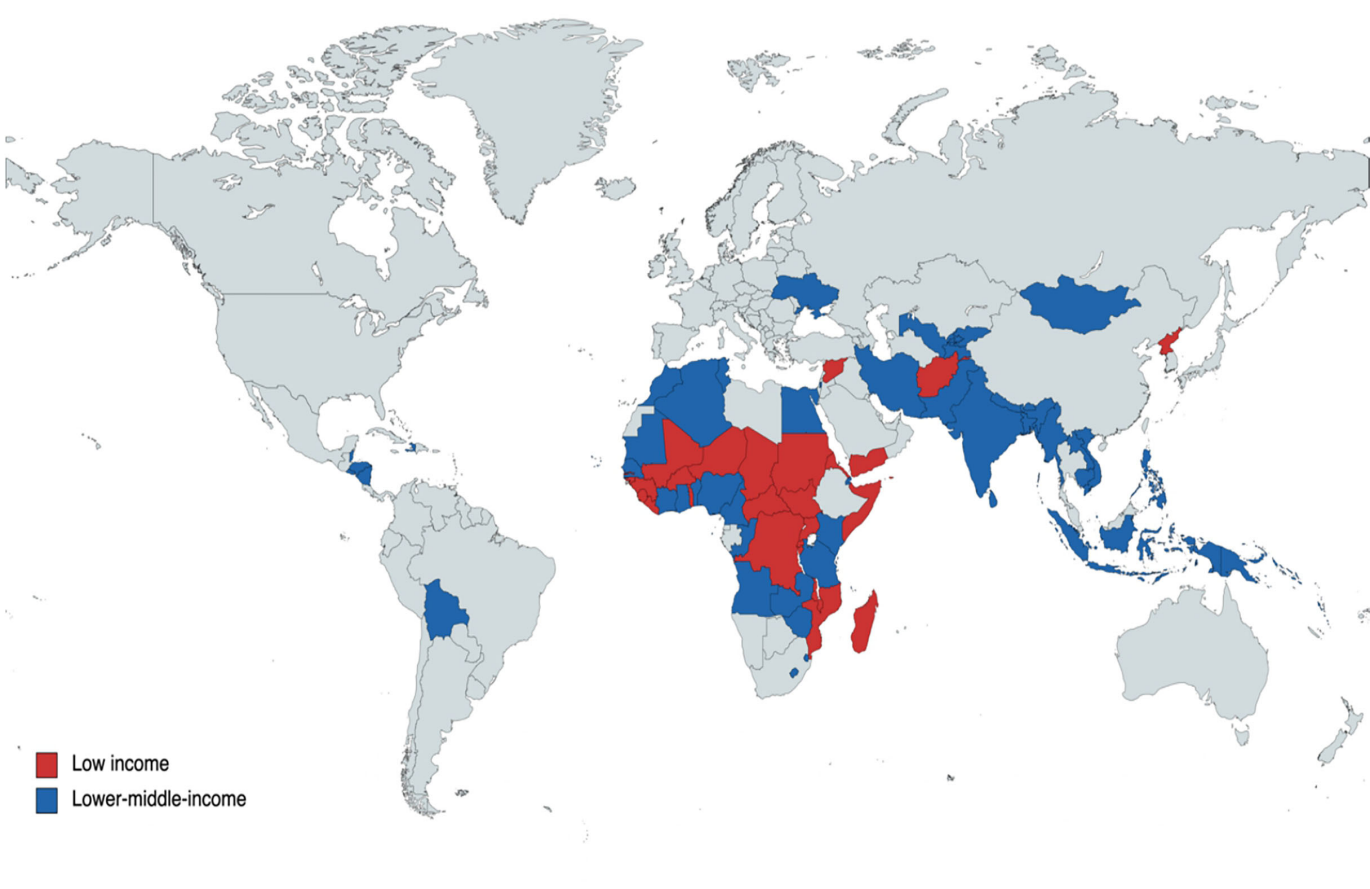
Distribution of low- and lower-middle-income countries across the world.

This scoping review sourced for studies between January 2002 and March 2022 that reported on ML-based models for cancer-related outcomes in low- or lower-middle-income countries (LLMICs). The composition of LLMICs was according to the 2022 World Bank classification of countries by income with 27 and 55 countries in the low and lower-middle income groups respectively (**Figure 1**) (22). ML-based models considered were those constructed on the backend of traditional machine learning and deep learning algorithms. Also, cancer-related outcomes cuts across risk stratification, screening, diagnosis, treatment selection, treatment complications, and prognosis of any malignancy which were assessed exclusively among LLMIC populations. Original retrospective or prospective studies were included in the review provided: (i) they were conducted exclusively among local populations in LLMICs for all model implementation phases, (ii) the outcomes were assessed among clinical patients or tumors linked to clinical patients, (iii) they featured an ML-based model/platform/software for which internal validation of models had been performed upon development (Phases I and II studies) (23).

Studies reporting multinational cohorts (even if they included LLMICs) or those that used LLMIC populations only for external validation were excluded. Further excluded, were studies featuring models constructed on public datasets or studies for which the development of the models among LLMICs was not described or referenced. However, for public datasets obtained from LLMIC populations, we only included the first study where the cohorts were initially recruited and analyzed for model construction. Studies with suspected data fabrication/falsification, incomplete reporting to assess development/validation population and ML modeling, and unclear stratification of malignant or high-risk classes (in the case of screening and risk stratification) were not selected. Duplicate studies (i.e., studies that used different models for the same outcome on a particular patient cohort), *in-vitro* studies, animal studies, commentaries, and editorials were also excluded. For duplicate reports, only the study on the models with the best performance was considered in this review.

Articles were sourced from PubMed, Medline, Web of Science, and Scopus electronic databases using nonspecific but pertinent combinations of search keywords for complete retrieval of related texts (**Supplementary Table 1**). Automated and manual deduplication of citations were then performed. We adopted a two-stage strategy for article selection. In one stage, we removed articles unrelated to the review objectives, studies based on inferential statistical associations, and those not comprising LLMIC populations. Subsequently, full-length texts of screened articles were obtained and assessed twice for each study according to the inclusion and exclusion criteria. These selection stages were performed by two independent authors (JA and AA) and discrepancies were resolved by consensus following discussions and agreement between both reviewers was the basis for the final study selection. During the second-stage selection, a complementary manual search of the references and citations of studies was performed to facilitate the inclusion of studies that may have been missed during electronic database searching. The agreement between both reviewers in selecting articles was near perfect with a κ value of 0.907 (p < 0.001) (19).

Data charting was done using full texts of selected articles. Items sourced included the author names, publication year, study LLMIC, study design, sample size, cancer types, outcomes, ML-model type, model development approach, features, implementation phases (23), and performance measures. For models with multiple validation populations, the mean of their accuracies was calculated and utilized in this study. Data collection was performed independently and in duplicate with the original full texts referred to for discordant entries.

Risk of bias assessment and quality rating of selected studies were performed using the Prediction model Risk of Bias Assessment tool (PROBAST) (24). All four original risk of bias domains were retained and scored as high, low, or unclear based on the total signaling questions per domain. The overall risk of bias ratings was given as ‘high’ if at least one domain was rated as ‘high/unclear’ and ‘low’ if all four domains were rated as ‘low’.

Qualitative synthesis was performed at the level of the studies and models for all LLMICs found. Assumptions were not made for missing data. Descriptive statistics were calculated as median, interquartile range, and frequencies. Pearson’s Chi-square test was used to determine significant differences for categorical variables while Kruskal-Wallis and Mann-Whitney U tests were used for continuous variables. Probability values below 5% were considered statistically significant. SPSS v 27 was used for all analyses. Reporting of this review was in line with the Preferred Reporting Items for Systematic Reviews and Meta-analysis extension for scoping reviews (PRISMA-ScR) (25) guidelines and the protocol registered with the International prospective register of systematic reviews (PROSPERO) with registration number CRD42022308345.

## Results

**Figure 2:**
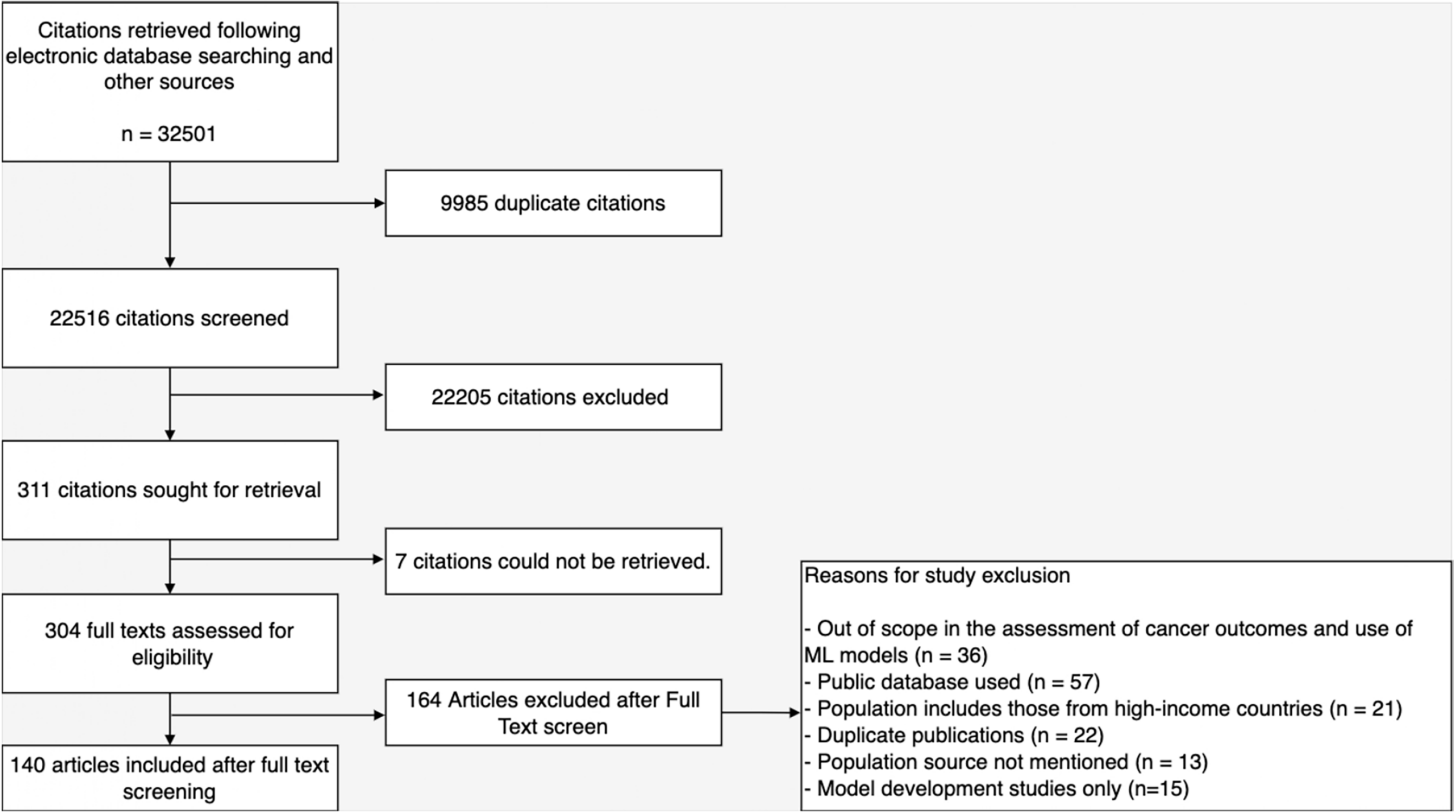
Flow chart of screening and study selection processes.

Upon removal of duplicates, 22,516 citations were screened leaving 311 articles for full-text evaluation. Altogether, 171 articles were excluded for reasons presented in **Figure 2** and 140 articles were included in this review. These articles emanated from 133 studies as model development and validation were reported in two separate articles by three studies while two studies reported their model construction in three different articles. Likewise, a total of 137 models for various cancer-related outcomes in LLMICs were extracted from these studies (26–165). Citations of included articles are listed in **Supplementary Table 2**.

### General characteristics of studies

**Figure 3:**
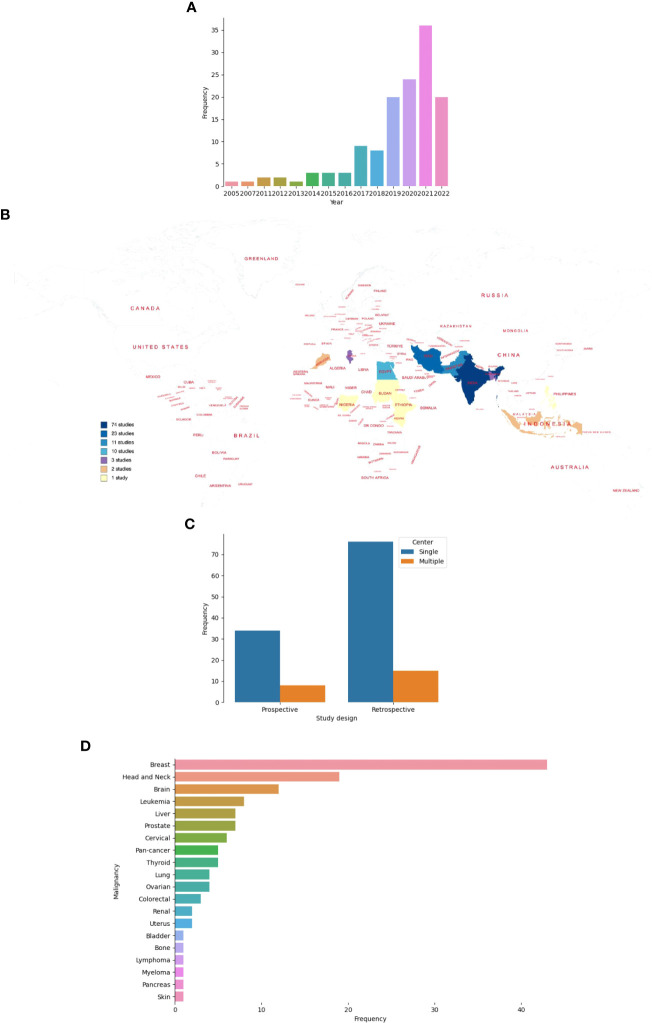
Characteristics of studies on ML-based models for cancer outcomes in LLMICs. **(A)** Bar plot showing the frequency of studies by publication year **(B)** Geographic distribution of studies on ML-models for cancer in LLMICs by country **(C)** Bar plot showing the study design and number of centers involved during model construction **(D)** Plot showing the frequency of cancers for which models were developed in the LLMIC population.

Studies were published between 2005 and 2022, and of 133 studies, 81.2% were published from 2018 onward (**Figure 3A**) (26–165). Of the 82 countries that make up LLMICs in this review, only 13 (15.8%) had at least a study included in this review (**Figure 3B**). All but two studies from Ethiopia and Sudan were conducted in lower-middle-income countries. Majority of the studies were from Indian (55.6%) and Iranian (17.3%) populations while four sub-Saharan African countries and the Philippines had only one study included. Irrespective of the number of centers involved, retrospective cohorts were often used for model constructions than prospective cohorts (68.4% vs 31.6%; **Figure 3C**). At least, outcomes from 19 different cancer types were modeled. However, five studies included patients with different cancer types. Only two studies involved patients with sarcomas (osteosarcoma and uterine sarcoma) while other studies involved those with carcinomas, leukemias, and lymphomas. Breast cancer (32.3%), head and neck cancer (14.3%), and brain tumors (9%) were the three most common malignancies by subtypes (**Figure 3D**). Further, we noticed a strong agreement between common malignancies used for ML modeling and the three most-incident tumors [based on GLOBOCAN estimates 2020 (1)] in each LLMIC represented (κ = 0.833, p=0.003).

### Risk of bias assessment for studies involving ML-based models in LLMICs

**Figure 4:**
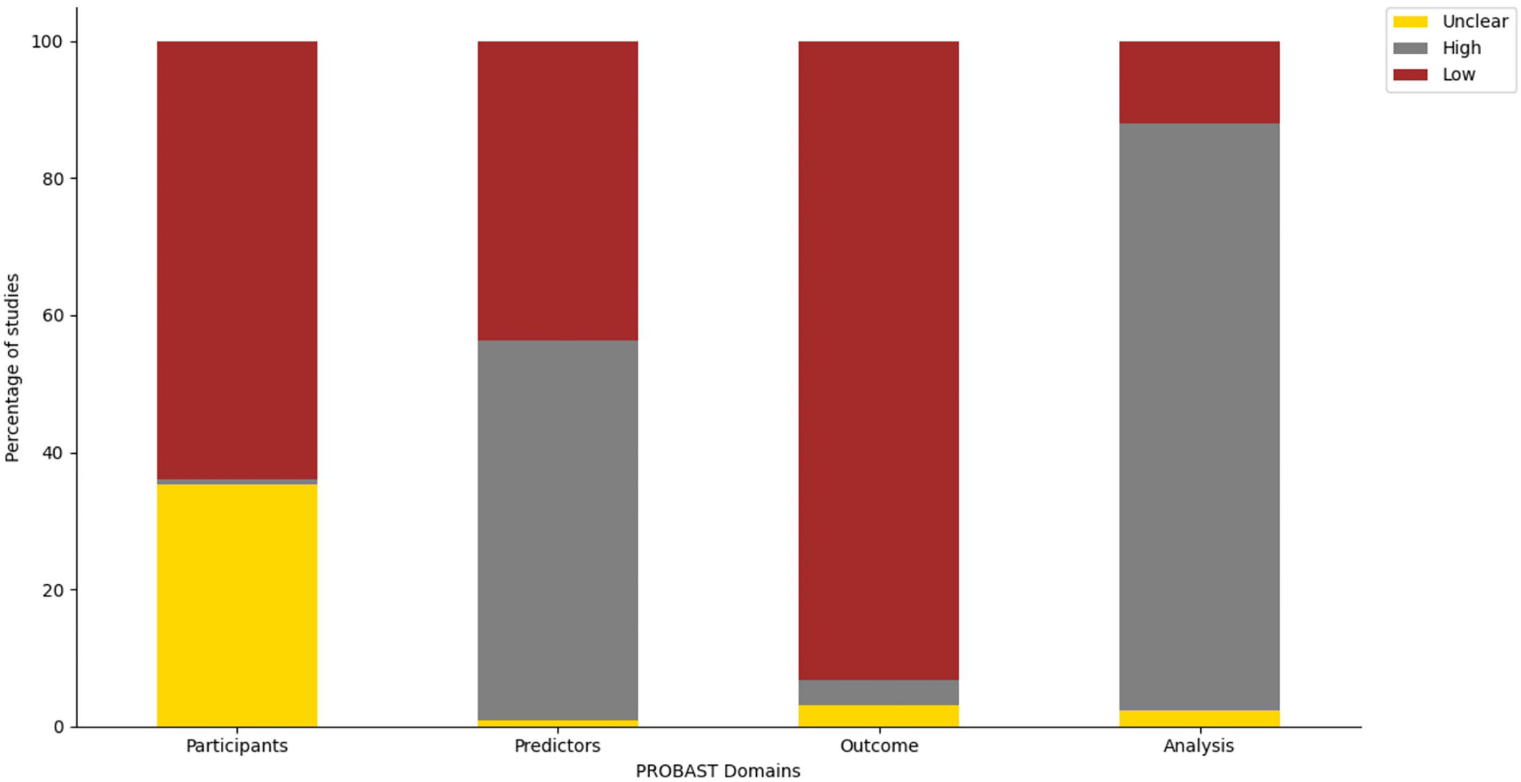
Distribution of studies by the PROBAST domains.

Only four studies (3.0%) had a low overall risk of bias across all four domains of the PROBAST tool (**Supplementary Table 3**). Proportion of studies with high and low risk of bias in each domain are plotted in **Figure 4**. More ML studies (93.2%) had a low risk of bias in their evaluation of outcomes while a majority of studies (85.7%) had a high risk of bias regarding the analytical methodology and technical approach to model construction.

Of the 114 studies with a high risk of bias in the ‘analysis’ domain, most studies did not have an event-per-variable (EPV) of at least 20 during training or at least 100 patients in each outcome category during model validation (n = 96). Moreover, other studies did not perform any data resampling methods (cross-validation, bootstrapping, or jackknife) using the training cohort to assess model stability or did not implement imbalanced class correction for model outcomes when necessitated. Due to the retrospective cohort design of many ML studies for cancers, predictors for cross-sectional outcomes such as diagnosis and treatment selection were selected while the outcome of the patients was known leading to their high risk of bias rating in the ‘predictors’ domain. Of note, 35.3% of studies had an unclear risk of bias in the ‘participants’ domain due to gross under-reporting of the inclusion and exclusion criteria used in the selection of patients for which outcomes were evaluated.

### Evaluating the methods of ML-based model construction for cancer outcomes in LLMIC populations

**Figure 5:**
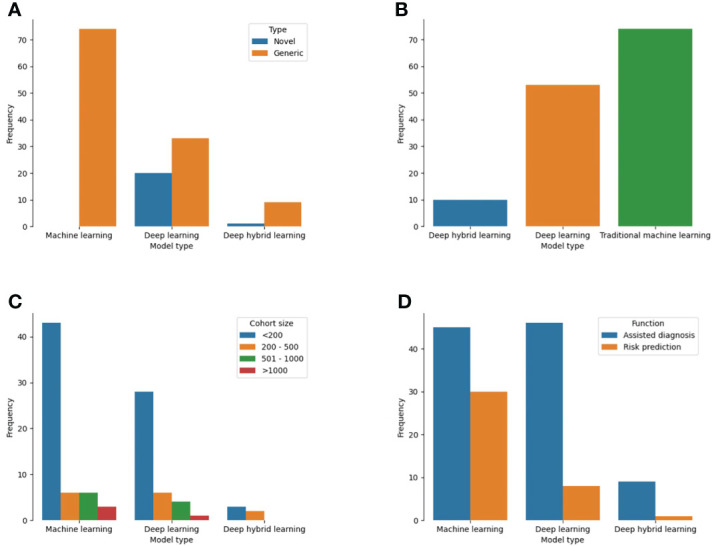
Plot showing the different techniques used for ML-based model construction for cancer patients in LLMICs **(A)** Model types **(B)** Novelty of backend algorithm used for model construction **(C)** Patient cohort size for model construction **(D)** Functions of the ML-based models.

Of 137 models, 74 (54%) were developed on the backend of traditional machine learning algorithms while 53 (38.6%) were based on deep learning (**Figure 5A**). Ten studies utilized both methods as a deep hybrid learning platform. Novel architectures were used for the development of 21 models (15.3%) which were mostly deep learning (95.2%) than deep hybrid or traditional machine learning models (**Figure 5B**; p<0.001). For studies that reported datasets used by the number of patients (n = 102), cohort sizes ranged from 10 to 5025 patients. A decreasing trend in the proportion of studies was noted with an increase in the number of patients used for model construction. More models were based on predictive features obtained from and for <200 patients (72.6%) than 200 to 500 (13.7%) or 500 to 1000 patients (9.8%). Only four models utilized 1000 patients or more. Stratifying the cohort size by the different ML techniques, all model types mostly involved < 200 patients (**Figure 5C**). Sample size estimation for model training and validation was only performed for 4 models (2.9%) (**Supplementary Table 4**).

Single cancer-related outcomes were considered in 134 models including cancer diagnosis (70.1%), overall prognosis/treatment response (14.6%), screening (8.0%), and treatment (5.1%). For the three models with multiple outcomes, two considered both cancer screening and diagnosis while one was used for tumor diagnosis and prognosis prediction (**Supplementary Table 4**). Tasks for which models were designed to perform included assisted diagnosis (70.8%), risk prediction/stratification (26.3%), and assisted treatment selection (0.7%). These functions were significantly different among the three types of ML techniques used for model construction as more traditional ML models than deep learning and deep hybrid learning (40% vs 14.8% vs 10%) were developed for risk prediction/stratification (**Figure 4D**, p = 0.003). Similarly, deep learning (85.2%) and deep hybrid learning (90%) models were mostly fashioned to assist in cancer diagnosis.

**Figure 6:**
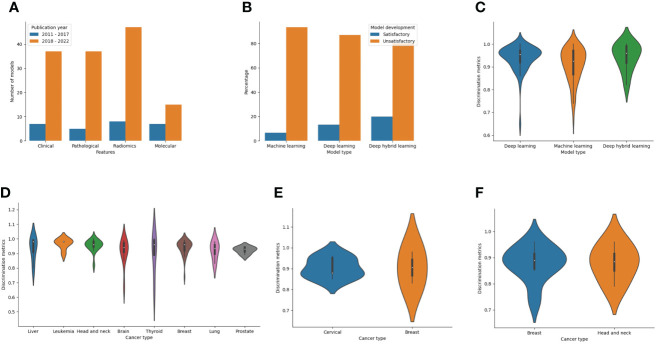
Bar and violin plots evaluating the methods used for model development and summary model performances **(A)** Plot of features used for model construction by publication year **(B)** Plot of ML technique by the rating of model development processes **(C)** Plot showing summary accuracy estimates for the three different ML techniques **(D)** Plot showing summary accuracy for ML-based models for diagnosis according to the cancer types **(E)** Plot showing summary accuracy for ML-based models for cancer screening according to the cancer types **(F)** Plot showing summary accuracy for ML-based models for cancer prognosis according to the cancer types.

Features used for model development among LLMIC populations were radiomics (40.2%), clinical (33.6%), pathological (30.7%), and molecular (16.1%) in nature. Of the radiomics datasets, 29 models used magnetic resonance imaging, 15 models utilized computed tomography scans, 10 models used mammograms, and 5 models used ultrasound scans (**Supplementary Table 4**). Also, biomarkers used as molecular features were proteomic (n=16), genomic (n=3), or metabolomic (n=3) in nature (**Supplementary Table 4**). Clinical and radiomic features were commonly used for ML model construction for cancer outcomes in LLMICs before 2018 while more recent years have seen higher incorporation of pathological and molecular features (**Figure 6A**). As realistic models for cancer outcomes often involve multidimensional features, only 23 models (16.8%) used at least two of the four feature categories with the combinations of clinical, pathological, and molecular variables being more common.

Comparison of different algorithms was often conducted before optimal model selection (106 models [77.4%]) and many models were evaluated alongside other ML models (75.2%) than traditional statistical models (13.1%). Of 107 models in which performance comparisons were done, five studies (4.7%) found that the models selected or optimized had an equivalent performance compared to others employed while one ML-based model was reported to have displayed no additional benefit in predictive performance following model comparison (**Supplementary Table 4**). Other ML-based models (93.5%) were deemed to have positive findings which motivated their selection for proposed tasks.

Considering the model development strategies underwent during construction, 123 of 137 models (89.8%) were deemed unsatisfactory in their approach. This was mostly due to a combination of inadequate sampling and improper technique (47.2%) than inadequate sampling (37.7%) or improper techniques (14.8%) alone (**Supplementary Table 4**). Common shortfalls in modeling techniques included the use of test-train splitting without nested cross-validation or bootstrapping during model training, not using feature selection even in the event of a low EPV, and lack of data augmentation or imbalance class correction when warranted. The lack of imbalanced class correction points to a limitation in the outcome class parity which may mean that the quality of data used for modeling is low. Although not statistically significant, stratifying the models based on the techniques showed that a higher proportion of deep hybrid models were satisfactory than deep learning or traditional ML models (20% vs 13.2% vs 6.8%, p=0.274) (**Figure 6B**). Also, according to the publication year, 1 of 25 models (4%) published before 2018 was satisfactory while for studies within the last five years, 13 of 112 models (11.6%) were satisfactory.

Of 124 models that were constructed after the establishment of the Transparent reporting of a multivariate prediction model for individual prognosis or diagnosis (TRIPOD) guideline or Standards for reporting diagnostic accuracy (STARD) guideline, only one model (79) followed the STARD guideline for model and performance reporting (**Supplementary Table 4**). None of the other models alluded to have followed any established guidelines during construction.

### Status of implementation, performance, and clinical impact of ML models in LLMICs

Generally, 128 (93.4%) models have completed phase I (data pre-preprocessing) and II (development and internal validation) implementation strategies only. Further, only 6 models (4.4%) constructed for cancer outcomes in LLMIC populations have been externally validated (phase III implementation) all of which were within the last five years. These included models for breast cancer diagnosis (85, 86, 152), lung cancer diagnosis (51, 52), head and neck cancer diagnosis (124), breast cancer metastasis (47, 56, 128), liver cancer risk prediction (78), and treatment response in colorectal cancer (148). Of these models, only two (47, 56, 85, 152) sufficiently fulfilled the TRIPOD criteria for external validation based on the sample size. Also, none of the deep learning or deep hybrid learning models found have been assessed using external validation.

Software for end-user interactions (phase IV implementation) has been designed for four ML-based models; however, three of these models were not even validated in an independent population before software development. Only one independently-validated ML-based model for breast cancer diagnosis before software development has also had a clinical impact assessment performed (phase V implementation). However, this was conducted using an observational study design (85).

The overall discriminatory performance (AUC/Accuracy) of the ML-based cancer models in LLMICs ranged from 0.65 – 1.00 for 134 models with a median (IQR) discriminatory performance of 0.94 (0.89 – 0.97). Both models based on deep hybrid learning and deep learning had median discrimination metrics (IQR) of 0.96 (0.90 – 0.99) and 0.96 (0.92 – 0.97) which was significantly higher than the median discrimination metrics of traditional ML models 0.93 (0.86 - 0.97) (**Figure 6C**; p = 0.041). Median accuracies according to the different cancer subtypes and outcomes (cancer screening, diagnosis, and prognosis) from at least three different models are shown in **Figures 6D–F**. For tumor diagnosis, while most median discrimination metrics were high, performance was highest for the use of ML models in leukemia diagnosis (0.96 [0.92 – 0.98]) than others. Summary discrimination for the incorporation of ML in cancer screening was higher for breast than cervical cancer screening while using ML-based models among LLMIC populations for breast cancer and head and neck cancer prognostication yielded an equivalent median accuracy of 0.89.

## Discussion

### Utilization of ML-based models for cancer outcomes in LLMICs

Assessing the methodology and implementation status of ML-based models and platforms for cancer outcomes in LLMICs is crucial to improving the prevention, diagnosis, and treatment of cancer in developing nations considering a predicted rise in disease incidence and mortality. This review found a recent increase in the construction of ML-based models for cancers among LLMIC populations that is in line with the general surge of ML application worldwide (166–168). However, intelligent model applications are chiefly limited to some lower-middle-income countries like India, Iran, Pakistan, and Egypt than others. Our study showed that ML-based models were not being applied for cancer prediction among indigenous populations in about 70 countries with a majority of these found to be within sub-Saharan Africa. This observation is likely inseparable from the overall challenges in health information technology and data management that preclude effective medical data accrual of indigenous populations within these countries (19, 169, 170). This reason is further bolstered by the wide use of public databases for ML modeling of cancer outcomes from authors affiliated with many lower-middle-income countries (19). Other reasons for the low popularity of ML-based models in many LLMICs may include the lack of expertise, personnel, and resources required to generate ML-based cancer models and a lack of awareness by stakeholders on the optimal ability of AI tools in predictive and classification health tasks compared to traditional methods (171, 172).

### Evaluating data and ML model construction for cancer outcomes in LLMICs

While standard ML algorithms were mostly used for model construction of cancer outcomes, the application of deep learning and deep hybrid learning models were found to be increasing in LLMICs. Of note, the quality of techniques performed during ML model development as well as the overall risk of bias for the studies was largely unsatisfactory. Though many models had excellent discriminatory performance in this study, this translates that many of these models are not currently streamlined enough to be considered for pivotal evaluation of their clinical impact in cancer management in LLMICs. The suboptimal models in this review were found to be a culmination of one of the following challenges: model overfitting, reduced cohort size, not incorporating methods to augment data or correct outcome imbalance, lack of model stability assessment, and cessation of further model validity assessment using external cohorts (23, 24, 173, 174). It is recommended that future studies involving ML-based models for cancer outcomes in LLMICs consider estimating sufficient cohort sizes for model development and validation while considering dimensionality reduction as well as data augmentation/balancing techniques in the event of reduced patient availability (175–177). Furthermore, other shortfalls may be addressed by compliance with standard guidelines for reporting protocols and studies for clinical models and their performances including the IJMEDI (173), TRIPOD (174, 178), STARD (179), SPIRIT-AI (180), and CONSORT-AI (181) statements.

Data quality assessments such as label parity, noisy data identification, outlier detection, and multicollinearity were sparsely assessed by the ML models. However, assessment of class parity based on the need for correction of imbalanced data which could be deduced from the modeling approach showed that this was infrequently performed when needed in the models. Considering the cohort sizes and the lack of class imbalance correction, this suggests that the quality of data used for ML modeling in LLMICs is low. However, this cannot be verified in the models given that other measures of data quality were not intentionally assessed in this study. This represents an additional task to be conducted in future ML models for cancer outcomes in LLMICs. For structured data, authors of ML-based models should consider the using of opensource quality assessment APIs such as the Data Quality Toolkit (182).

### Degree of Implementation of ML-based models for cancer outcomes in LLMICs

Regarding the cancer types for which outcomes were modeled using ML algorithms, breast cancer and head and neck cancer (mostly oral cancer) were the most common in line with the majority of LLMICs represented in this review (1). However, outcomes for some cancers uniformly common in many developing countries and have infectious causes related to poverty and awareness such as Kaposi’s sarcoma, Burkitt’s lymphoma, and gastric cancer have not been considered (1, 183–185). Hopefully, with an increase in cancer ML-based model development among more LLMICs in the future, outcomes for these specific malignancies will be further explored for indigenous patients. Likewise, the future should see an increasing application of ML-based models for other cancers associated with low socioeconomic status like cervical and liver cancer (1).

Only a few models in this review have been optimized for software development and even rare was the impact assessment of this software. Of note, none of the models developed for cancer outcomes among LLMIC populations have been evaluated for clinical efficacy and impact in a randomized controlled trial. This is unsurprising given the recency in which model development and performance were performed. However, stakeholders in LLMICs should ensure that ML-based models for cancer outcomes undergo rigorous evaluation in this manner before clinical implementation if applicable (19, 23, 186). Also, challenges to implementation other than model performance including settings for applications, exact application in cancer care, data standardization, and availability of features for prediction should be assessed during these rigorous evaluations.

### Limitations

The findings of this review should be interpreted while considering the following limitations: (i) Our search strategy may have missed studies not indexed in the popular electronic databases used in this study. However, the search was limited to these databases to ensure the inclusion of mostly high-quality studies which were not presented in predatory journals (ii) Articles from LLMICs in satisfactory indigenous journals only available in print forms or reported in national languages other than English may have also been missed (iii) Due to the large number of studies included in the scoping review, the characteristics of individual studies was not provided within the main text but presented in **Supplementary Table 4** (iv) This study recognizes that the challenges identified regarding the application of ML-based models for cancer outcomes in LLMICs may not be specific to these areas. Therefore, future studies should focus on comparing the objectives of this review across regions and settings of different income categories.

## Conclusions

A recent increase in the application of ML-based prevention and intervention for cancer in LLMICs was observed in this study. This surge was also associated with only little improvement in the implementation progress of ML-based models in a few of these countries. Generally, ML-based models were mostly generated among certain lower-middle-income countries like India, Iran, Pakistan, and Egypt than others and were chiefly employed for diagnostic outcomes. This showcases the need to intensify model development and/or external validation endeavors in all low-income countries and many lower-middle-income countries, especially within sub-Saharan Africa. Overall, given the suboptimal methods used for the development of existing models, their lack of external validation and clinical impact assessments, and the lack of adherence to standard guidelines, many ML-based models for cancer outcomes among indigenous patients in LLMICs are not presently streamlined enough for pivotal evaluation and clinical application. We recommend model retraining using larger sample sizes and intensified external validation practices for ML-based models developed for cancer among LLMIC populations.

## Data availability statement

The original contributions presented in the study are included in the article/**Supplementary Material**. Further inquiries can be directed to the corresponding author.

## Author contributions

JA conceptualized the study, performed the search, study selection, risk of bias assessment, and wrote the original draft of the manuscript. AA assisted in study concepts, study selection, risk of bias assessment, and manuscript review and editing. MK-M was involved in study methodology, interpretation of results, machine learning modelling consultations, and manuscript review and editing. PT and Y-XS contributed to the interpretation of results, critical review of the manuscript, and provided supervision. All authors contributed to the article and approved the submitted version.

## Conflict of interest

The authors declare that the research was conducted in the absence of any commercial or financial relationships that could be construed as a potential conflict of interest.

## Publisher’s note

All claims expressed in this article are solely those of the authors and do not necessarily represent those of their affiliated organizations, or those of the publisher, the editors and the reviewers. Any product that may be evaluated in this article, or claim that may be made by its manufacturer, is not guaranteed or endorsed by the publisher.
