# Supplementary material for: Construction of machine learning-based models for cancer outcomes in low and lower-middle income countries: A scoping review
Source: Front Oncol. 2022 Dec 1;12:976168. doi: 10.3389/fonc.2022.976168 (PMC9751812; doi:10.3389/fonc.2022.976168)
Supplement: Supplementary file 1 [file Table_1.docx]

**Supplementary Table 1**

Search Keywords and operationalization in two databases

| Key words | Machine learning, machine intelligence, algorithms, machine models, artificial intelligence, deep learning, neural networks, machine classifiers, cancer, oncology, malignancy, neoplasms |
| --- | --- |
| PubMed/Medline | (machine learning OR machine intelligence OR artificial intelligence OR deep learning OR natural language processing OR machine classifiers OR neural network OR machine algorithms OR machine models) AND (cancer OR oncology OR malignancy OR neoplasms) |
| Web of science | (machine learning OR machine intelligence OR artificial intelligence OR deep learning OR natural language processing OR machine classifiers OR neural network OR machine algorithms OR machine models) AND (cancer OR oncology OR malignancy OR neoplasms) |
| Scopus | (machine learning OR machine intelligence OR artificial intelligence OR deep learning OR natural language processing OR machine classifiers OR neural network OR machine algorithms OR machine models) AND (cancer OR oncology OR malignancy OR neoplasms) |

**Supplementary Table 2**

| **SN** | **STUDIES** |
| --- | --- |
|  | Aatresh, A. A., Alabhya, K., Lal, S., Kini, J., & Saxena, P. U. P. (2021). LiverNet: efficient and robust deep learning model for automatic diagnosis of sub-types of liver hepatocellular carcinoma cancer from H&E stained liver histopathology images. *Int J Comput Assist Radiol Surg*, *16*(9), 1549-1563. https://doi.org/10.1007/s11548-021-02410-4 |
|  | Abdollahi, H., Mahdavi, S. R., Mofid, B., Bakhshandeh, M., Razzaghdoust, A., Saadipoor, A., & Tanha, K. (2018). Rectal wall MRI radiomics in prostate cancer patients: prediction of and correlation with early rectal toxicity. *Int J Radiat Biol*, *94*(9), 829-837. https://doi.org/10.1080/09553002.2018.1492756 |
|  | Abdollahi, H., Mofid, B., Shiri, I., Razzaghdoust, A., Saadipoor, A., Mahdavi, A., Galandooz, H. M., & Mahdavi, S. R. (2019). Machine learning-based radiomic models to predict intensity-modulated radiation therapy response, Gleason score and stage in prostate cancer. *Radiol Med*, *124*(6), 555-567. https://doi.org/10.1007/s11547-018-0966-4 |
|  | Abdollahi, H., Mostafaei, S., Cheraghi, S., Shiri, I., Rabi Mahdavi, S., & Kazemnejad, A. (2018). Cochlea CT radiomics predicts chemoradiotherapy induced sensorineural hearing loss in head and neck cancer patients: A machine learning and multi-variable modelling study. *Phys Med*, *45*, 192-197. https://doi.org/10.1016/j.ejmp.2017.10.008 |
|  | Acharya, V., Ravi, V., Pham, T. D., & Chakraborty, C. Peripheral Blood Smear Analysis Using Automated Computer-Aided Diagnosis System to Identify Acute Myeloid Leukemia. *Ieee Transactions on Engineering Management*. https://doi.org/10.1109/tem.2021.3103549 |
|  | Agarwal, P., Yadav, A., Mathur, P., Pal, V., & Chakrabarty, A. (2022). BID-Net: An Automated System for Bone Invasion Detection Occurring at Stage T4 in Oral Squamous Carcinoma Using Deep Learning. *Comput Intell Neurosci*, *2022*, 4357088. https://doi.org/10.1155/2022/4357088 |
|  | Ahammed Muneer, K. V., Rajendran, V. R., & K, P. J. (2019). Glioma Tumor Grade Identification Using Artificial Intelligent Techniques. *J Med Syst*, *43*(5), 113. https://doi.org/10.1007/s10916-019-1228-2 |
|  | Ahmed, J., & Rehman, M. A. (2016). CANCER PREVENTION INITIATIVE: AN INTELLIGENT APPROACH FOR THYROID CANCER TYPE DIAGNOSTICS. *Jurnal Teknologi*, *78*(4-3), 31-39. |
|  | Al Amin, A., Parvin, S., Kadir, M. A., Tahmid, T., Alam, S. K., & Siddique-e Rabbani, K. (2014). Classification of breast tumour using electrical impedance and machine learning techniques. *Physiol Meas*, *35*(6), 965-974. https://doi.org/10.1088/0967-3334/35/6/965 |
|  | Al-Dhabyani, W., Fahmy, A., Gomaa, M., & Khaled, H. (2019). Deep Learning Approaches for Data Augmentation and Classification of Breast Masses using Ultrasound Images. *International Journal of Advanced Computer Science and Applications*, *10*(5), 618-627. <Go to ISI>://WOS:000476620600079 |
|  | Ali, M., Sarwar, A., Sharma, V., & Suri, J. (2019). Artificial neural network based screening of cervical cancer using a hierarchical modular neural network architecture (HMNNA) and novel benchmark uterine cervix cancer database. *Neural Computing & Applications*, *31*(7), 2979-2993. https://doi.org/10.1007/s00521-017-3246-7 |
|  | Allahloubi, N. M. A., Zekri, A. R. N., Ragab, M., Mohanad, M., Ahmed, O. S., Eid, S., Ghareeb, M., Gouda, I., & Bahnassy, A. A. Estrogen Receptor Gene Polymorphism as a Possible Genetic Risk Factor for Treatment Response in ER-Positive Breast Cancer Patients. *Biochemical Genetics*. https://doi.org/10.1007/s10528-022-10199-3 |
|  | Amin, J., Sharif, M., Raza, M., Saba, T., & Anjum, M. A. (2019). Brain tumor detection using statistical and machine learning method. *Comput Methods Programs Biomed*, *177*, 69-79. https://doi.org/10.1016/j.cmpb.2019.05.015 |
|  | Amiri, S., Akbarabadi, M., Abdolali, F., Nikoofar, A., Esfahani, A. J., & Cheraghi, S. (2021). Radiomics analysis on CT images for prediction of radiation-induced kidney damage by machine learning models. *Comput Biol Med*, *133*, 104409. https://doi.org/10.1016/j.compbiomed.2021.104409 |
|  | Ara, S. R., Alam, F., Rahman, M. H., Akhter, S., Awwal, R., & Hasan, K. (2015). Bimodal Multiparameter-Based Approach for Benign-Malignant Classification of Breast Tumors. *Ultrasound Med Biol*, *41*(7), 2022-2038. https://doi.org/10.1016/j.ultrasmedbio.2015.01.023 |
|  | Asaduzzaman, S., Ahmed, M. R., Rehana, H., Chakraborty, S., Islam, M. S., & Bhuiyan, T. (2021). Machine learning to reveal an astute risk predictive framework for Gynecologic Cancer and its impact on women psychology: Bangladeshi perspective. *BMC Bioinformatics*, *22*(1), 213. https://doi.org/10.1186/s12859-021-04131-6 |
|  | Assari, Z., Mahloojifar, A., & Ahmadinejad, N. (2022). A bimodal BI-RADS-guided GoogLeNet-based CAD system for solid breast masses discrimination using transfer learning. *Computers in Biology and Medicine*, *142*. https://doi.org/10.1016/j.compbiomed.2021.105160 |
|  | Asuntha, A., & Srinivasan, A. (2020). Deep learning for lung Cancer detection and classification. *Multimedia Tools and Applications*, *79*(11-12), 7731-7762. https://doi.org/10.1007/s11042-019-08394-3 |
|  | Aswiga, R. V., Aishwarya, R., & Shanthi, A. P. (2021). Augmenting Transfer Learning with Feature Extraction Techniques for Limited Breast Imaging Datasets. *J Digit Imaging*, *34*(3), 618-629. https://doi.org/10.1007/s10278-021-00456-z |
|  | Ayyad, S. M., Badawy, M. A., Shehata, M., Alksas, A., Mahmoud, A., Abou El-Ghar, M., Ghazal, M., El-Melegy, M., Abdel-Hamid, N. B., Labib, L. M., Ali, H. A., & El-Baz, A. (2022). A New Framework for Precise Identification of Prostatic Adenocarcinoma. *Sensors (Basel)*, *22*(5). https://doi.org/10.3390/s22051848 |
|  | Babu, T., Singh, T., Gupta, D., & Hameed, S. (2021). Colon cancer prediction on histological images using deep learning features and Bayesian optimized SVM. *Journal of Intelligent & Fuzzy Systems*, *41*(5), 5275-5286. https://doi.org/10.3233/jifs-189850 |
|  | Bakre, M. M., Ramkumar, C., Attuluri, A. K., Basavaraj, C., Prakash, C., Buturovic, L., Madhav, L., Naidu, N., R, P., Somashekhar, S. P., Gupta, S., Doval, D. C., & Pegram, M. D. (2019). Clinical validation of an immunohistochemistry-based CanAssist-Breast test for distant recurrence prediction in hormone receptor-positive breast cancer patients. *Cancer Med*, *8*(4), 1755-1764. https://doi.org/10.1002/cam4.2049 |
|  | Bal, A., Das, M., Satapathy, S. M., Jena, M., & Das, S. K. (2021). BFCNet: a CNN for diagnosis of ductal carcinoma in breast from cytology images. *Pattern Analysis and Applications*, *24*(3), 967-980. https://doi.org/10.1007/s10044-021-00962-4 |
|  | Ben Youssef, Y., Abdelmounim, E., Zbitou, J., Elharoussi, M., & Boujida, M. N. (2017). Comparison Machine Learning Algorithms in Abnormal Mammograms Classification. *International Journal of Computer Science and Network Security*, *17*(5), 19-25. <Go to ISI>://WOS:000412566800003 |
|  | Bhowmik, A., Ghosh, B., Pal, M., Paul, R. R., Chatterjee, J., & Chakraborty, S. (2022). Portable, handheld, and affordable blood perfusion imager for screening of subsurface cancer in resource-limited settings. *Proc Natl Acad Sci U S A*, *119*(2). https://doi.org/10.1073/pnas.2026201119 |
|  | Binson, V. A., Subramoniam, M., & Mathew, L. (2021). Noninvasive detection of COPD and Lung Cancer through breath analysis using MOS Sensor array based e-nose. *Expert Review of Molecular Diagnostics*, *21*(11), 1223-1233. https://doi.org/10.1080/14737159.2021.1971079 |
|  | Binson, V. A., Subramoniam, M., Sunny, Y., & Mathew, L. (2021). Prediction of Pulmonary Diseases With Electronic Nose Using SVM and XGBoost. *Ieee Sensors Journal*, *21*(18), 20886-20895. https://doi.org/10.1109/jsen.2021.3100390 |
|  | Buddhavarapu, V. G., & Jothi, J. A. A. (2020). An experimental study on classification of thyroid histopathology images using transfer learning. *Pattern Recognition Letters*, *140*, 1-9. https://doi.org/10.1016/j.patrec.2020.09.020 |
|  | Bukhari, S. U. K., Bokhari, S. K. A., Syed, A., Hussain, S. S., Armaghan, S. U., & Shah, S. S. H. (2020). The Diagnostic Accuracy of Convolutional Neural Network Architectures for the Diagnosis of Brain Cancer. *Pakistan Journal of Medical & Health Sciences*, *14*(3), 1037-1039. <Go to ISI>://WOS:000619502700150 |
|  | Butola, A., Ahmad, A., Dubey, V., Srivastava, V., Qaiser, D., Srivastava, A., Senthilkumaran, P., & Mehta, D. S. (2019). Volumetric analysis of breast cancer tissues using machine learning and swept-source optical coherence tomography. *Appl Opt*, *58*(5), A135-a141. https://doi.org/10.1364/ao.58.00a135 |
|  | Chandra Doval, D., Mehta, A., Somashekhar, S. P., Gunda, A., Singh, G., Bal, A., Khare, S., Prakash, V. S. C., Adinarayan, M., Krishnamoorthy, N., Vijay, D. G., Anantakrishnan, R., Bhattacharyya, G. S., & Bakre, M. M. (2021). The usefulness of CanAssist breast in the assessment of recurrence risk in patients of ethnic Indian origin. *Breast*, *59*, 1-7. https://doi.org/10.1016/j.breast.2021.05.007 |
|  | Chandra Joshi, R., Mishra, R., Gandhi, P., Pathak, V. K., Burget, R., & Dutta, M. K. (2021). Ensemble based machine learning approach for prediction of glioma and multi-grade classification. *Comput Biol Med*, *137*, 104829. https://doi.org/10.1016/j.compbiomed.2021.104829 |
|  | Daoud, B., Morooka, K., Kurazume, R., Leila, F., Mnejja, W., & Daoud, J. (2019). 3D segmentation of nasopharyngeal carcinoma from CT images using cascade deep learning. *Comput Med Imaging Graph*, *77*, 101644. https://doi.org/10.1016/j.compmedimag.2019.101644 |
|  | Das, A., Acharya, U. R., Panda, S. S., & Sabut, S. (2019). Deep learning based liver cancer detection using watershed transform and Gaussian mixture model techniques. *Cognitive Systems Research*, *54*, 165-175. https://doi.org/10.1016/j.cogsys.2018.12.009 |
|  | Das, D., & Mahanta, L. B. (2021). A Comparative Assessment of Different Approaches of Segmentation and Classification Methods on Childhood Medulloblastoma Images. *Journal of Medical and Biological Engineering*, *41*(3), 379-392. https://doi.org/10.1007/s40846-021-00612-4 |
|  | Das, D. K., Mitra, P., Chakraborty, C., Chatterjee, S., Maiti, A. K., & Bose, S. (2017). Computational approach for mitotic cell detection and its application in oral squamous cell carcinoma. *Multidimensional Systems and Signal Processing*, *28*(3), 1031-1050. https://doi.org/10.1007/s11045-017-0488-6 |
|  | Das, N., Hussain, E., & Mahanta, L. B. (2020). Automated classification of cells into multiple classes in epithelial tissue of oral squamous cell carcinoma using transfer learning and convolutional neural network. *Neural Netw*, *128*, 47-60. https://doi.org/10.1016/j.neunet.2020.05.003 |
|  | Dese, K., Raj, H., Ayana, G., Yemane, T., Adissu, W., Krishnamoorthy, J., & Kwa, T. (2021). Accurate Machine-Learning-Based classification of Leukemia from Blood Smear Images. *Clin Lymphoma Myeloma Leuk*, *21*(11), e903-e914. https://doi.org/10.1016/j.clml.2021.06.025 |
|  | Devakumar, D., Sunny, G., Sasidharan, B. K., Bowen, S. R., Nadaraj, A., Jeyseelan, L., Mathew, M., Irodi, A., Isiah, R., Pavamani, S., John, S., & Thomas, H. M. T. (2021). Framework for Machine Learning of CT and PET Radiomics to Predict Local Failure after Radiotherapy in Locally Advanced Head and Neck Cancers. *Journal of Medical Physics*, *46*(3), 181-188. https://doi.org/10.4103/jmp.JMP_6_21 |
|  | Dheeba, J., Jaya, T., & Singh, N. A. (2017). Breast cancer risk assessment and diagnosis model using fuzzy support vector machine based expert system. *Journal of Experimental & Theoretical Artificial Intelligence*, *29*(5), 1011-1021. https://doi.org/10.1080/0952813x.2017.1280088 |
|  | El Agouri, H., Azizi, M., El Attar, H., El Khannoussi, M., Ibrahimi, A., Kabbaj, R., Kadiri, H., BekarSabein, S., EchCharif, S., Mounjid, C., & El Khannoussi, B. (2022). Assessment of deep learning algorithms to predict histopathological diagnosis of breast cancer: first Moroccan prospective study on a private dataset. *BMC Res Notes*, *15*(1), 66. https://doi.org/10.1186/s13104-022-05936-1 |
|  | Fathi, E., Rezaee, M. J., Tavakkoli-Moghaddam, R., Alizadeh, A., & Montazer, A. (2020). Design of an integrated model for diagnosis and classification of pediatric acute leukemia using machine learning. *Proc Inst Mech Eng H*, *234*(10), 1051-1069. https://doi.org/10.1177/0954411920938567 |
|  | Fathi Kazerooni, A., Nabil, M., Zeinali Zadeh, M., Firouznia, K., Azmoudeh-Ardalan, F., Frangi, A. F., Davatzikos, C., & Saligheh Rad, H. (2018). Characterization of active and infiltrative tumorous subregions from normal tissue in brain gliomas using multiparametric MRI. *J Magn Reson Imaging*, *48*(4), 938-950. https://doi.org/10.1002/jmri.25963 |
|  | Ferjaoui, R., Cherni, M. A., Boujnah, S., Kraiem, N. E. H., & Kraiem, T. (2021). Machine learning for evolutive lymphoma and residual masses recognition in whole body diffusion weighted magnetic resonance images. *Comput Methods Programs Biomed*, *209*, 106320. https://doi.org/10.1016/j.cmpb.2021.106320 |
|  | Figueroa, K. C., Song, B., Sunny, S., Li, S., Gurushanth, K., Mendonca, P., Mukhia, N., Patrick, S., Gurudath, S., Raghavan, S., Imchen, T., Leivon, S. T., Kolur, T., Shetty, V., Bushan, V., Ramesh, R., Pillai, V., Wilder-Smith, P., Sigamani, A., . . . Liang, R. (2022). Interpretable deep learning approach for oral cancer classification using guided attention inference network. *J Biomed Opt*, *27*(1). https://doi.org/10.1117/1.Jbo.27.1.015001 |
|  | Gangil, T., Shahabuddin, A. B., Rao, B. D., Palanisamy, K., Chakrabarti, B., & Sharan, K. (2022). Predicting clinical outcomes of radiotherapy for head and neck squamous cell carcinoma patients using machine learning algorithms. *Journal of Big Data*, *9*(1), Article 25. https://doi.org/10.1186/s40537-022-00578-3 |
|  | Gehlot, S., Gupta, A., & Gupta, R. (2021). A CNN-based unified framework utilizing projection loss in unison with label noise handling for multiple Myeloma cancer diagnosis. *Med Image Anal*, *72*, 102099. https://doi.org/10.1016/j.media.2021.102099 |
|  | Giri, S., Bose, J. C., Chandrasekar, A., Tiwary, B. K., Gajalakshmi, P., & Chatterjee, S. (2019). Increased Plasma Nitrite and von Willebrand Factor Indicates Early Diagnosis of Vascular Diseases in Chemotherapy Treated Cancer Patients. *Cardiovasc Toxicol*, *19*(1), 36-47. https://doi.org/10.1007/s12012-018-9471-1 |
|  | Gnanasekaran, V. S., Joypaul, S., Sundaram, P. M., & Chairman, D. D. (2020). Deep learning algorithm for breast masses classification in mammograms. *Iet Image Processing*, *14*(12), 2860-2868. https://doi.org/10.1049/iet-ipr.2020.0070 |
|  | Gollapalli, K., Ray, S., Srivastava, R., Renu, D., Singh, P., Dhali, S., Bajpai Dikshit, J., Srikanth, R., Moiyadi, A., & Srivastava, S. (2012). Investigation of serum proteome alterations in human glioblastoma multiforme. *Proteomics*, *12*(14), 2378-2390. https://doi.org/10.1002/pmic.201200002 |
|  | Gupta, M., Gupta, A., Yadav, V., Parvaze, S. P., Singh, A., Saini, J., Patir, R., Vaishya, S., Ahlawat, S., & Gupta, R. K. (2021). Comparative evaluation of intracranial oligodendroglioma and astrocytoma of similar grades using conventional and T1-weighted DCE-MRI. *Neuroradiology*, *63*(8), 1227-1239. https://doi.org/10.1007/s00234-021-02636-8 |
|  | Hammouda, K., Khalifa, F., Soliman, A., Ghazal, M., El-Ghar, M. A., Badawy, M. A., Darwish, H. E., Khelifi, A., & El-Baz, A. (2021). A multiparametric MRI-based CAD system for accurate diagnosis of bladder cancer staging. *Comput Med Imaging Graph*, *90*, 101911. https://doi.org/10.1016/j.compmedimag.2021.101911 |
|  | Hashem, S., ElHefnawi, M., Habashy, S., El-Adawy, M., Esmat, G., Elakel, W., Abdelazziz, A. O., Nabeel, M. M., Abdelmaksoud, A. H., Elbaz, T. M., & Shousha, H. I. (2020). Machine Learning Prediction Models for Diagnosing Hepatocellular Carcinoma with HCV-related Chronic Liver Disease. *Comput Methods Programs Biomed*, *196*, 105551. https://doi.org/10.1016/j.cmpb.2020.105551 |
|  | Holmström, O., Linder, N., Kaingu, H., Mbuuko, N., Mbete, J., Kinyua, F., Törnquist, S., Muinde, M., Krogerus, L., Lundin, M., Diwan, V., & Lundin, J. (2021). Point-of-Care Digital Cytology With Artificial Intelligence for Cervical Cancer Screening in a Resource-Limited Setting. *JAMA Netw Open*, *4*(3), e211740. https://doi.org/10.1001/jamanetworkopen.2021.1740 |
|  | Hussain, E., Mahanta, L. B., Das, C. R., & Talukdar, R. K. (2020). A comprehensive study on the multi-class cervical cancer diagnostic prediction on pap smear images using a fusion-based decision from ensemble deep convolutional neural network. *Tissue Cell*, *65*, 101347. https://doi.org/10.1016/j.tice.2020.101347 |
|  | Jajodia, A., Gupta, A., Prosch, H., Mayerhoefer, M., Mitra, S., Pasricha, S., Mehta, A., Puri, S., & Chaturvedi, A. (2021). Combination of Radiomics and Machine Learning with Diffusion-Weighted MR Imaging for Clinical Outcome Prognostication in Cervical Cancer. *Tomography*, *7*(3), 344-357. https://doi.org/10.3390/tomography7030031 |
|  | Jajroudi, M., Enferadi, M., Homayoun, A. A., & Reiazi, R. (2022). MRI-based machine learning for determining quantitative and qualitative characteristics affecting the survival of glioblastoma multiforme. *Magnetic Resonance Imaging*, *85*, 222-227. https://doi.org/10.1016/j.mri.2021.10.023 |
|  | James, B. L., Sunny, S. P., Heidari, A. E., Ramanjinappa, R. D., Lam, T., Tran, A. V., Kankanala, S., Sil, S., Tiwari, V., Patrick, S., Pillai, V., Shetty, V., Hedne, N., Shah, D., Shah, N., Chen, Z. P., Kandasarma, U., Raghavan, S. A., Gurudath, S., . . . Kuriakose, M. A. (2021). Validation of a Point-of-Care Optical Coherence Tomography Device with Machine Learning Algorithm for Detection of Oral Potentially Malignant and Malignant Lesions. *Cancers*, *13*(14), Article 3583. https://doi.org/10.3390/cancers13143583 |
|  | Jha, S., & Mehta, A. K. (2022). An evolutionary algorithm based feature selection and fuzzy rule reduction technique for the prediction of skin cancer. *Concurrency and Computation-Practice & Experience*, *34*(5), Article e6694. https://doi.org/10.1002/cpe.6694 |
|  | Kakileti, S. T., Madhu, H. J., Krishnan, L., Manjunath, G., Sampangi, S., & Ramprakash, H. V. (2020). Observational Study to Evaluate the Clinical Efficacy of Thermalytix for Detecting Breast Cancer in Symptomatic and Asymptomatic Women. *JCO Glob Oncol*, *6*, 1472-1480. https://doi.org/10.1200/go.20.00168 |
|  | Kakileti, S. T., Madhu, H. J., Manjunath, G., Wee, L., Dekker, A., & Sampangi, S. (2020). Personalized risk prediction for breast cancer pre-screening using artificial intelligence and thermal radiomics. *Artif Intell Med*, *105*, 101854. https://doi.org/10.1016/j.artmed.2020.101854 |
|  | Kamath, S. D., & Mahato, K. K. (2007). Optical pathology using oral tissue fluorescence spectra: classification by principal component analysis and k-means nearest neighbor analysis. *J Biomed Opt*, *12*(1), 014028. https://doi.org/10.1117/1.2437738 |
|  | Kamath, S. D., Ray, S., & Mahato, K. K. (2011). Photoacoustic spectroscopy of ovarian normal, benign, and malignant tissues: a pilot study. *J Biomed Opt*, *16*(6), 067001. https://doi.org/10.1117/1.3583573 |
|  | Kansal, S., Goel, S., Bhattacharya, J., & Srivastava, V. (2020). Generative adversarial network-convolution neural network based breast cancer classification using optical coherence tomographic images. *Laser Physics*, *30*(11), Article 115601. https://doi.org/10.1088/1555-6611/abb596 |
|  | Kashef, A., Khatibi, T., & Mehrvar, A. (2020). Prediction of Cranial Radiotherapy Treatment in Pediatric Acute Lymphoblastic Leukemia Patients Using Machine Learning: A Case Study at MAHAK Hospital. *Asian Pac J Cancer Prev*, *21*(11), 3211-3219. https://doi.org/10.31557/apjcp.2020.21.11.3211 |
|  | Kaur, I., Doja, M. N., & Ahmad, T. (2020). Time-range based sequential mining for survival prediction in prostate cancer. *J Biomed Inform*, *110*, 103550. https://doi.org/10.1016/j.jbi.2020.103550 |
|  | Kaur, I., Doja, M. N., Ahmad, T., Ahmad, M., Hussain, A., Nadeem, A., & Abd El-Latif, A. A. (2021). An Integrated  Approach for Cancer Survival Prediction Using Data Mining Techniques. *Comput Intell Neurosci*, *2021*, 6342226. https://doi.org/10.1155/2021/6342226 |
|  | Kaur, R., Juneja, M., & Mandal, A. K. (2021). Machine learning based quantitative texture analysis of CT images for diagnosis of renal lesions. *Biomedical Signal Processing and Control*, *64*, Article 102311. https://doi.org/10.1016/j.bspc.2020.102311 |
|  | Kaushik, M., Chandra Joshi, R., Kushwah, A. S., Gupta, M. K., Banerjee, M., Burget, R., & Dutta, M. K. (2021). Cytokine gene variants and socio-demographic characteristics as predictors of cervical cancer: A machine learning approach. *Comput Biol Med*, *134*, 104559. https://doi.org/10.1016/j.compbiomed.2021.104559 |
|  | Kayal, E. B., Kandasamy, D., Sharma, R., Bakhshi, S., & Mehndiratta, A. (2020). Segmentation of osteosarcoma tumor using diffusion weighted MRI: a comparative study using nine segmentation algorithms. *Signal Image and Video Processing*, *14*(4), 727-735. https://doi.org/10.1007/s11760-019-01599-x |
|  | Khan, S. U., Islam, N., Jan, Z., Din, I. U., Khan, A., & Faheem, Y. (2019). An e-Health care services framework for the detection and classification of breast cancer in breast cytology images as an IoMT application. *Future Generation Computer Systems-the International Journal of Escience*, *98*, 286-296. https://doi.org/10.1016/j.future.2019.01.033 |
|  | Lal, S., Das, D., Alabhya, K., Kanfade, A., Kumar, A., & Kini, J. (2021). NucleiSegNet: Robust deep learning architecture for the nuclei segmentation of liver cancer histopathology images. *Comput Biol Med*, *128*, 104075. https://doi.org/10.1016/j.compbiomed.2020.104075 |
|  | Lotfnezhad Afshar, H., Jabbari, N., Khalkhali, H. R., & Esnaashari, O. (2021). Prediction of Breast Cancer Survival by Machine Learning Methods: An Application of Multiple Imputation. *Iranian Journal of Public Health*, *50*(3), 598-605. |
|  | Macaulay, B. O., Aribisala, B. S., Akande, S. A., Akinnuwesi, B. A., & Olabanjo, O. A. (2021). Breast cancer risk prediction in African women using Random Forest Classifier. *Cancer Treat Res Commun*, *28*, 100396. https://doi.org/10.1016/j.ctarc.2021.100396 |
|  | Mahanta, L. B., Hussain, E., Das, N., Kakoti, L., & Chowdhury, M. (2021). IHC-Net: A fully convolutional neural network for automated nuclear segmentation and ensemble classification for Allred scoring in breast pathology. *Applied Soft Computing*, *103*, Article 107136. https://doi.org/10.1016/j.asoc.2021.107136 |
|  | Mahmood, N., Shahid, S., Bakhshi, T., Riaz, S., Ghufran, H., & Yaqoob, M. (2020). Identification of significant risks in pediatric acute lymphoblastic leukemia (ALL) through machine learning (ML) approach. *Med Biol Eng Comput*, *58*(11), 2631-2640. https://doi.org/10.1007/s11517-020-02245-2 |
|  | Mahmood, T., Li, J., Pei, Y., Akhtar, F., Rehman, M. U., & Wasti, S. H. (2022). Breast lesions classifications of mammographic images using a deep convolutional neural network-based approach. *PLoS One*, *17*(1), e0263126. https://doi.org/10.1371/journal.pone.0263126 |
|  | Majumder, B., Baraneedharan, U., Thiyagarajan, S., Radhakrishnan, P., Narasimhan, H., Dhandapani, M., Brijwani, N., Pinto, D. D., Prasath, A., Shanthappa, B. U., Thayakumar, A., Surendran, R., Babu, G. K., Shenoy, A. M., Kuriakose, M. A., Bergthold, G., Horowitz, P., Loda, M., Beroukhim, R., Majumder, P. K. (2015). Predicting clinical response to anticancer drugs using an ex vivo platform that captures tumour heterogeneity. *Nat Commun*, *6*, 6169. https://doi.org/10.1038/ncomms7169 |
|  | Majumder, S. K., Ghosh, N., & Gupta, P. K. (2005). Relevance vector machine for optical diagnosis of cancer. *Lasers Surg Med*, *36*(4), 323-333. https://doi.org/10.1002/lsm.20160 |
|  | Malek, M., Gity, M., Alidoosti, A., Oghabian, Z., Rahimifar, P., Seyed Ebrahimi, S. M., Tabibian, E., & Oghabian, M. A. (2019). A machine learning approach for distinguishing uterine sarcoma from leiomyomas based on perfusion weighted MRI parameters. *Eur J Radiol*, *110*, 203-211. https://doi.org/10.1016/j.ejrad.2018.11.009 |
|  | Malek, M., Tabibian, E., Rahimi Dehgolan, M., Rahmani, M., Akhavan, S., Sheikh Hasani, S., Nili, F., & Hashemi, H. (2020). A Diagnostic Algorithm using Multi-parametric MRI to Differentiate Benign from Malignant Myometrial Tumors: Machine-Learning Method. *Sci Rep*, *10*(1), 7404. https://doi.org/10.1038/s41598-020-64285-w |
|  | Mathialagan, P., & Chidambaranathan, M. (2021). Computer vision techniques for Upper Aero-Digestive Tract tumor grading classification-Addressing pathological challenges. *Pattern Recognition Letters*, *144*, 42-53. https://doi.org/10.1016/j.patrec.2021.01.002 |
|  | Mohamed N, van de Goor R, El-Sheikh M, Elrayah O, Osman T, Nginamau ES, Johannessen AC, Suleiman A, Costea DE, Kross KW. Feasibility of a Portable Electronic Nose for Detection of Oral Squamous Cell Carcinoma in Sudan. Healthcare (Basel). 2021;9(5). |
|  | Mohebian, M. R., Marateb, H. R., Mansourian, M., Mananas, M. A., & Mokarian, F. (2017). A Hybrid Computer-aided-diagnosis System for Prediction of Breast Cancer Recurrence (HPBCR) Using Optimized Ensemble Learning. *Computational and Structural Biotechnology Journal*, *15*, 75-85. https://doi.org/10.1016/j.csbj.2016.11.004 |
|  | Mokni, R., Gargouri, N., Damak, A., Sellami, D., Feki, W., & Mnif, Z. (2021). An automatic Computer-Aided Diagnosis system based on the Multimodal fusion of Breast Cancer (MF-CAD). *Biomedical Signal Processing and Control*, *69*, Article 102914. https://doi.org/10.1016/j.bspc.2021.102914 |
|  | Montazeri, M., Montazeri, M., Montazeri, M., & Beigzadeh, A. (2016). Machine learning models in breast cancer survival prediction. *Technol Health Care*, *24*(1), 31-42. https://doi.org/10.3233/thc-151071 |
|  | Mostafaei, S., Abdollahi, H., Dehkordi, S. K., Shiri, I., Razzaghdoust, A., Moghaddam, S. H. Z., Saadipoor, A., Koosha, F., Cheraghi, S., & Mahdavi, S. R. (2020). CT imaging markers to improve radiation toxicity prediction in prostate cancer radiotherapy by stacking regression algorithm. *Radiologia Medica*, *125*(1), 87-97. https://doi.org/10.1007/s11547-019-01082-0 |
|  | Mungle, T., Tewary, S., Das, D. K., Arun, I., Basak, B., Agarwal, S., Ahmed, R., Chatterjee, S., & Chakraborty, C. (2017). MRF-ANN: a machine learning approach for automated ER scoring of breast cancer immunohistochemical images. *J Microsc*, *267*(2), 117-129. https://doi.org/10.1111/jmi.12552 |
|  | Nachimuthu, D. S., & Baladhandapani, A. (2014). Multidimensional texture characterization: on analysis for brain tumor tissues using MRS and MRI. *J Digit Imaging*, *27*(4), 496-506. https://doi.org/10.1007/s10278-013-9669-5 |
|  | Naeem, S., Ali, A., Qadri, S., Mashwani, W. K., Tairan, N., Shah, H., Fayaz, M., Jamal, F., Chesneau, C., & Anam, S. (2020). Machine-Learning Based Hybrid-Feature Analysis for Liver Cancer Classification Using Fused (MR and CT) Images. *Applied Sciences-Basel*, *10*(9), Article 3134. https://doi.org/10.3390/app10093134 |
|  | Nanditha, B. R., Kiran, A. G., Chandrashekar, H. S., Dinesh, M. S., & Murali, S. (2021). An Ensemble Deep Neural Network Approach for Oral Cancer Screening. *International Journal of Online and Biomedical Engineering*, *17*(2), 121-134. https://doi.org/10.3991/ijoe.v17i02.19207 |
|  | Nassar, A., Lymona, A. M., Lotfy, M. M., Youssef, A. S. E., Mohanad, M., Manie, T. M., Youssef, M. M. G., Farahat, I. G., & Zekri, A. N. (2021). Tumor Mutation Burden Prediction Model in Egyptian Breast Cancer patients based on Next Generation Sequencing. *Asian Pac J Cancer Prev*, *22*(7), 2053-2059. https://doi.org/10.31557/apjcp.2021.22.7.2053 |
|  | Naushad, S. M., Dorababu, P., Rupasree, Y., Pavani, A., Raghunadharao, D., Hussain, T., Alrokayan, S. A., & Kutala, V. K. (2019). Classification and regression tree-based prediction of 6-mercaptopurine-induced leucopenia grades in children with acute lymphoblastic leukemia. *Cancer Chemother Pharmacol*, *83*(5), 875-880. https://doi.org/10.1007/s00280-019-03803-8 |
|  | Nawandhar, A., Kumar, N., Veena, R., & Yamujala, L. (2020). Stratified squamous epithelial biopsy image classifier using machine learning and neighborhood feature selection. *Biomedical Signal Processing and Control*, *55*, Article 101671. https://doi.org/10.1016/j.bspc.2019.101671 |
|  | Nayak, A., Baidya Kayal, E., Arya, M., Culli, J., Krishan, S., Agarwal, S., & Mehndiratta, A. (2019). Computer-aided diagnosis of cirrhosis and hepatocellular carcinoma using multi-phase abdomen CT. *Int J Comput Assist Radiol Surg*, *14*(8), 1341-1352. https://doi.org/10.1007/s11548-019-01991-5 |
|  | Negahbani, F., Sabzi, R., Pakniyat Jahromi, B., Firouzabadi, D., Movahedi, F., Kohandel Shirazi, M., Majidi, S., & Dehghanian, A. (2021). PathoNet introduced as a deep neural network backend for evaluation of Ki-67 and tumor-infiltrating lymphocytes in breast cancer. *Sci Rep*, *11*(1), 8489. https://doi.org/10.1038/s41598-021-86912-w |
|  | Nisa, M., Buzdar, S. A., Khan, K., & Ahmad, M. S. (2022). Deep Convolutional Neural Network Based Analysis of Liver Tissues Using Computed Tomography Images. *Symmetry-Basel*, *14*(2), Article 383. https://doi.org/10.3390/sym14020383 |
|  | Panigrahi, S., Bhuyan, R., Kumar, K., Nayak, J., & Swarnkar, T. (2022). Multistage classification of oral histopathological images using improved residual network. *Math Biosci Eng*, *19*(2), 1909-1925. https://doi.org/10.3934/mbe.2022090 |
|  | Paul, A., Srivastava, S., Roy, R., Anand, A., Gaurav, K., Husain, N., Jain, S., & Sonkar, A. A. (2020). Malignancy prediction among tissues from Oral SCC patients including neck invasions: a (1)H HRMAS NMR based metabolomic study. *Metabolomics*, *16*(3), 38. https://doi.org/10.1007/s11306-020-01660-8 |
|  | Paydar, S., Pourahmad, S., Azad, M., Bolandparvaz, S., Taheri, R., Ghahramani, Z., Zamani, A., Jeddi, M., Karimi, F., Dabbaghmanesh, M. H., Shams, M., & Abbasi, H. R. (2016). The Evolution of a Malignancy Risk Prediction Model for Thyroid Nodules Using the Artificial Neural Network. *Middle East Journal of Cancer*, *7*(1), 47-52. <Go to ISI>://WOS:000379600200006 |
|  | Punithavathy, K., Poobal, S., & Ramya, M. M. (2019). Performance Evaluation of Machine Learning Techniques in Lung Cancer Classification from PET/CT Images. *Fme Transactions*, *47*(3), 418-423. https://doi.org/10.5937/fmet1903418P |
|  | Rahman, T. Y., Mahanta, L. B., Das, A. K., & Sarma, J. D. (2020). Automated oral squamous cell carcinoma identification using shape, texture and color features of whole image strips. *Tissue Cell*, *63*, 101322. https://doi.org/10.1016/j.tice.2019.101322 |
|  | Ramkumar, C., Buturovic, L., Malpani, S., Attuluri, A. K., Basavaraj, C., Prakash, C., Madhav, L., Doval, D. C., Mehta, A., & Bakre, M. M. (2018). Development of a Novel Proteomic Risk-Classifier for Prognostication of Patients With Early-Stage Hormone Receptor-Positive Breast Cancer. *Biomarker Insights*, *13*, Article 1177271918789100. https://doi.org/10.1177/1177271918789100 |
|  | Ranjith, G., Parvathy, R., Vikas, V., Chandrasekharan, K., & Nair, S. (2015). Machine learning methods for the classification of gliomas: Initial results using features extracted from MR spectroscopy. *Neuroradiol J*, *28*(2), 106-111. https://doi.org/10.1177/1971400915576637 |
|  | Rashmi, R., Prasad, K., & Udupa, C. B. K. (2021). BCHisto-Net: Breast histopathological image classification by global and local feature aggregation. *Artif Intell Med*, *121*, 102191. https://doi.org/10.1016/j.artmed.2021.102191 |
|  | Reda, I., Khalil, A., Elmogy, M., Abou El-Fetouh, A., Shalaby, A., Abou El-Ghar, M., Elmaghraby, A., Ghazal, M., & El-Baz, A. (2018). Deep Learning Role in Early Diagnosis of Prostate Cancer. *Technol Cancer Res Treat*, *17*, 1533034618775530. https://doi.org/10.1177/1533034618775530 |
|  | Reda, I., Shalaby, A., Elmogy, M., Elfotouh, A. A., Khalifa, F., El-Ghar, M. A., Hosseini-Asl, E., Gimel'farb, G., Werghi, N., & El-Baz, A. (2017). A comprehensive non-invasive framework for diagnosing prostate cancer. *Comput Biol Med*, *81*, 148-158. https://doi.org/10.1016/j.compbiomed.2016.12.010 |
|  | Rehman, A., Abbas, N., Saba, T., Rahman, S. I. U., Mehmood, Z., & Kolivand, H. (2018). Classification of acute lymphoblastic leukemia using deep learning. *Microsc Res Tech*, *81*(11), 1310-1317. https://doi.org/10.1002/jemt.23139 |
|  | Rehman, K. U., Li, J. Q., Pei, Y., Yasin, A., Ali, S., & Saeed, Y. (2022). Architectural Distortion-Based Digital Mammograms Classification Using Depth Wise Convolutional Neural Network. *Biology-Basel*, *11*(1), Article 15. https://doi.org/10.3390/biology11010015 |
|  | Sadewo, W., Rustam, Z., Hamidah, H., & Chusmarsyah, A. R. (2020). Pancreatic Cancer Early Detection Using Twin Support Vector Machine Based on Kernel. *Symmetry-Basel*, *12*(4), Article 667. https://doi.org/10.3390/sym12040667 |
|  | Saha, M., Chakraborty, C., Arun, I., Ahmed, R., & Chatterjee, S. (2017). An Advanced Deep Learning Approach for Ki-67 Stained Hotspot Detection and Proliferation Rate Scoring for Prognostic Evaluation of Breast Cancer. *Sci Rep*, *7*(1), 3213. https://doi.org/10.1038/s41598-017-03405-5 |
|  | Saikia, A. R., Bora, K., Mahanta, L. B., & Das, A. K. (2019). Comparative assessment of CNN architectures for classification of breast FNAC images. *Tissue Cell*, *57*, 8-14. https://doi.org/10.1016/j.tice.2019.02.001 |
|  | Samuri, S. M., Nova, T. V., Rahmatullah, B., Li, W. S., & Al-Qaysi, Z. T. (2022). CLASSIFICATION MODEL FOR BREAST CANCER MAMMOGRAMS. *Iium Engineering Journal*, *23*(1), 187-199. https://doi.org/10.31436/iiumej.v23i1.1825 |
|  | Sanyal, P., Barui, S., Deb, P., & Sharma, H. C. (2019). Performance of A Convolutional Neural Network in Screening Liquid Based Cervical Cytology Smears. *Journal of Cytology*, *36*(3), 146-151. https://doi.org/10.4103/joc.Joc_201_18 |
|  | Sathya, D. J., & Geetha, K. (2013). Mass classification in breast DCE-MR images using an artificial neural network trained via a bee colony optimization algorithm. *Scienceasia*, *39*(3), 294-305. https://doi.org/10.2306/scienceasia1513-1874.2013.39.294 |
|  | Savala, R., Dey, P., & Gupta, N. (2018). Artificial neural network model to distinguish follicular adenoma from follicular carcinoma on fine needle aspiration of thyroid. *Diagn Cytopathol*, *46*(3), 244-249. https://doi.org/10.1002/dc.23880 |
|  | Sengupta, A., Ramaniharan, A. K., Gupta, R. K., Agarwal, S., & Singh, A. (2019). Glioma grading using a machine-learning framework based on optimized features obtained from T(1) perfusion MRI and volumes of tumor components. *J Magn Reson Imaging*, *50*(4), 1295-1306. https://doi.org/10.1002/jmri.26704 |
|  | Sengupta, D., Ali, S. N., Bhattacharya, A., Mustafi, J., Mukhopadhyay, A., & Sengupta, K. (2022). A deep hybrid learning pipeline for accurate diagnosis of ovarian cancer based on nuclear morphology. *PLoS One*, *17*(1), e0261181. https://doi.org/10.1371/journal.pone.0261181 |
|  | Sepandi, M., Taghdir, M., Rezaianzadeh, A., & Rahimikazerooni, S. (2018). Assessing Breast Cancer Risk with an Artificial Neural Network. *Asian Pac J Cancer Prev*, *19*(4), 1017-1019. https://doi.org/10.22034/apjcp.2018.19.4.1017 |
|  | Shaban, M., Khurram, S. A., Fraz, M. M., Alsubaie, N., Masood, I., Mushtaq, S., Hassan, M., Loya, A., & Rajpoot, N. M. (2019). A Novel Digital Score for Abundance of Tumour Infiltrating Lymphocytes Predicts Disease Free Survival in Oral Squamous Cell Carcinoma. *Sci Rep*, *9*(1), 13341. https://doi.org/10.1038/s41598-019-49710-z |
|  | Shaikh, T. A., Ali, R., & Beg, M. M. S. (2020). Transfer learning privileged information fuels CAD diagnosis of breast cancer. *Machine Vision and Applications*, *31*(1-2), Article 9. https://doi.org/10.1007/s00138-020-01058-5 |
|  | Sharafeldeen, A., Elsharkawy, M., Khaled, R., Shaffie, A., Khalifa, F., Soliman, A., Abdel Razek, A. A. K., Hussein, M. M., Taman, S., Naglah, A., Alrahmawy, M., Elmougy, S., Yousaf, J., Ghazal, M., & El-Baz, A. (2022). Texture and shape analysis of diffusion-weighted imaging for thyroid nodules classification using machine learning. *Med Phys*, *49*(2), 988-999. https://doi.org/10.1002/mp.15399 |
|  | Shayesteh, S., Nazari, M., Salahshour, A., Sandoughdaran, S., Hajianfar, G., Khateri, M., Yaghobi Joybari, A., Jozian, F., Fatehi Feyzabad, S. H., Arabi, H., Shiri, I., & Zaidi, H. (2021). Treatment response prediction using MRI-based pre-, post-, and delta-radiomic features and machine learning algorithms in colorectal cancer. *Med Phys*, *48*(7), 3691-3701. https://doi.org/10.1002/mp.14896 |
|  | Shayesteh, S. P., Alikhassi, A., Fard Esfahani, A., Miraie, M., Geramifar, P., Bitarafan-Rajabi, A., & Haddad, P. (2019). Neo-adjuvant chemoradiotherapy response prediction using MRI based ensemble learning method in rectal cancer patients. *Phys Med*, *62*, 111-119. https://doi.org/10.1016/j.ejmp.2019.03.013 |
|  | Shehata, M., Alksas, A., Abouelkheir, R. T., Elmahdy, A., Shaffie, A., Soliman, A., Ghazal, M., Abu Khalifeh, H., Salim, R., Abdel Razek, A. A. K., Alghamdi, N. S., & El-Baz, A. (2021). A Comprehensive Computer-Assisted Diagnosis System for Early Assessment of Renal Cancer Tumors. *Sensors (Basel)*, *21*(14). https://doi.org/10.3390/s21144928 |
|  | Shukla, R. S., & Aggarwal, Y. (2018). Nonlinear Heart Rate Variability based artificial intelligence in lung cancer prediction. *Journal of Applied Biomedicine*, *16*(2), 145-155. https://doi.org/10.1016/j.jab.2017.12.002 |
|  | Singh, A., Bhat, V., Sudhakar, S., Namachivayam, A., Gangadharan, C., Pulchan, C., & Sigamani, A. (2021). Multicentric study to evaluate the effectiveness of Thermalytix as compared with standard screening modalities in subjects who show possible symptoms of suspected breast cancer. *BMJ Open*, *11*(10), e052098. https://doi.org/10.1136/bmjopen-2021-052098 |
|  | Singh, B. K., Verma, K., Panigrahi, L., & Thoke, A. S. (2017). Integrating radiologist feedback with computer aided diagnostic systems for breast cancer risk prediction in ultrasonic images: An experimental investigation in machine learning paradigm. *Expert Systems with Applications*, *90*, 209-223. https://doi.org/10.1016/j.eswa.2017.08.020 |
|  | Singh, D., Kumar, V., Das, C. J., Singh, A., & Mehndiratta, A. (2021). Characterisation of prostate cancer using texture analysis for diagnostic and prognostic monitoring. *NMR Biomed*, *34*(6), e4495. https://doi.org/10.1002/nbm.4495 |
|  | Subbaiah, R. M., Dey, P., & Nijhawan, R. (2014). Artificial neural network in breast lesions from fine-needle aspiration cytology smear. *Diagn Cytopathol*, *42*(3), 218-224. https://doi.org/10.1002/dc.23026 |
|  | Tapak, L., Shirmohammadi-Khorram, N., Amini, P., Alafchi, B., Hamidi, O., & Poorolajal, J. (2019). Prediction of survival and metastasis in breast cancer patients using machine learning classifiers. *Clinical Epidemiology and Global Health*, *7*(3), 293-299. https://doi.org/10.1016/j.cegh.2018.10.003 |
|  | Tekchandani, H., Verma, S., Londhe, N. D., Jain, R. R., & Tiwari, A. (2022). Computer aided diagnosis system for cervical lymph nodes in CT images using deep learning. *Biomedical Signal Processing and Control*, *71*, Article 103158. https://doi.org/10.1016/j.bspc.2021.103158 |
|  | Tewary, S., Arun, I., Ahmed, R., Chatterjee, S., & Chakraborty, C. (2017). AutoIHC-scoring: a machine learning framework for automated Allred scoring of molecular expression in ER- and PR-stained breast cancer tissue. *J Microsc*, *268*(2), 172-185. https://doi.org/10.1111/jmi.12596 |
|  | Tewary, S., Arun, I., Ahmed, R., Chatterjee, S., & Mukhopadhyay, S. (2021). AutoIHC-Analyzer: computer-assisted microscopy for automated membrane extraction/scoring in HER2 molecular markers. *J Microsc*, *281*(1), 87-96. https://doi.org/10.1111/jmi.12955 |
|  | Thakran, S., Gupta, R. K., & Singh, A. Characterization of breast tumors using machine learning based upon multiparametric magnetic resonance imaging features. *Nmr in Biomedicine*, Article e4665. https://doi.org/10.1002/nbm.4665 |
|  | Tomas, R. C., Sayat, A. J., Atienza, A. N., Danganan, J. L., Ramos, M. R., Fellizar, A., Notarte, K. I., Angeles, L. M., Bangaoil, R., Santillan, A., & Albano, P. M. (2022). Detection of breast cancer by ATR-FTIR spectroscopy using artificial neural networks. *PLoS One*, *17*(1), e0262489. https://doi.org/10.1371/journal.pone.0262489 |
|  | Torkaman, A., Charkari, N. M., & Aghaeipour, M. (2011). An approach for leukemia classification based on cooperative game theory. *Anal Cell Pathol (Amst)*, *34*(5), 235-246. https://doi.org/10.3233/acp-2011-0016 |
|  | Vaka, A. R., Soni, B., & Reddy, K. S. (2020). Breast cancer detection by leveraging Machine Learning. *Ict Express*, *6*(4), 320-324. https://doi.org/10.1016/j.icte.2020.04.009 |
|  | Vazifehdan, M., Moattar, M. H., & Jalali, M. (2019). A hybrid Bayesian network and tensor factorization approach for missing value imputation to improve breast cancer recurrence prediction. *Journal of King Saud University-Computer and Information Sciences*, *31*(2), 175-184. https://doi.org/10.1016/j.jksuci.2018.01.002 |
|  | Zadeh, H. G., Pakdelazar, O., Haddadnia, J., Rezai-Rad, G., & Mohammad-Zadeh, M. (2012). Diagnosing Breast Cancer with the Aid of Fuzzy Logic Based on Data Mining of a Genetic Algorithm in Infrared Images. *Middle East Journal of Cancer*, *3*(4), 119-129. |
